# Guidance on providing telebehavioral health care for perinatal populations: An assessment of resources and gaps

**DOI:** 10.1007/s00737-026-01770-7

**Published:** 2026-09-23

**Authors:** Armenda Bialas, Julia Rollison, Jessica L. Sousa, Rachel K. Landis, Laura J. Faherty

**Affiliations:** 1 RAND Corporation, Santa Monica, USA; 2 Tufts University, Medford, USA

**Keywords:** Perinatal, Telehealth, Maternal health, Behavioral health, Implementation guidance

## Abstract

**Purpose:**

Perinatal behavioral health conditions are a leading cause of maternal morbidity and mortality. Telehealth can reduce access barriers, but availability of implementation guidance specific to the perinatal period is unknown.

**Methods:**

We conducted a comprehensive environmental scan of 71 organizational websites and nine semi-structured clinician interviews to identify clinician-facing telehealth implementation resources geared towards those practicing in the United States.

**Results:**

Twenty organizations (28%) published perinatal telehealth materials; sixteen provided nineteen unique resources specific to perinatal telebehavioral health. Most resources (*n* = 14) provided broad guidance relevant to the perinatal period, while six addressed prenatal telehealth care. No resources provided postpartum-specific guidance. Interviews revealed mixed perspectives on the adequacy of clinical guidance resources.

**Conclusion:**

Limited and outdated resources, particularly the absence of postpartum‑specific guidance, suggest the need for coordinated, perinatal‑specific telebehavioral health implementation materials.

## Introduction

Perinatal behavioral health conditions—including mood and anxiety disorders and substance use disorders—represent one of the most common complications affecting women during pregnancy and the first 12 months postpartum (American College of Obstetricians and Gynecologists n.d.-a; American Psychiatric Association 2023). Despite their prevalence, these conditions remain underdiagnosed and undertreated, and suicide is now the leading cause of death during the perinatal period, with more than a quarter of maternal deaths in the United States (U.S.) attributed to behavioral health conditions (Brown et al. 2021; Centers for Disease Control and Prevention 2026). This crisis disproportionately affects historically marginalized communities, mirroring broader inequities in maternal health outcomes (Mukherjee et al. 2016; Sujan et al. 2023). The treatment gap is substantial, with 11% of individuals experiencing perinatal mental health conditions receiving needed care, and even lower rates among marginalized populations who face additional screening and treatment barriers (Declercq et al. 2022; Huggins et al. 2020; Kozhimannil et al. 2011; Landis et al. 2023a, b; Sidebottom et al. 2021; Vesga-López et al. 2008).

Telehealth offers a promising opportunity to improve access and continuity of perinatal behavioral health care. Evidence demonstrates comparable outcomes to in-person care, while addressing many key access barriers including transportation difficulties, childcare-related responsibilities, and scheduling conflicts (Aboujaoude et al. 2015; Batastini et al. 2021; Hilty et al. 2013; Mohr et al. 2012). Beyond addressing these barriers, the provision of perinatal telebehavioral health care offers distinct advantages, including the ability to observe patients in their home environment, greater continuity across the pregnancy-to-postpartum transition, reduced no-show rates, and improved reach to rural and otherwise underserved populations. Yet implementing telehealth for this population requires tailored guidance that helps clinicians navigate rapidly shifting coverage policies (Center for Connected Health Policy 2024; Koonin et al. 2020) and unique clinical considerations in the perinatal period, such as hormonal and other physiologic changes, sleep disruption, and caregiving demands (Carlson et al. 2025; Kissler et al. 2024; Maternal Mental Health Leadership Alliance 2024; American College of Obstetricians and Gynecologists 2020).

We conducted an environmental scan of publicly available U.S.-based telebehavioral health implementation resources and interviewed clinicians to identify existing materials and highlight guidance deficits that may hinder effective care delivery for perinatal populations.

## Materials and methods

We conducted a systematic inventory of publicly available implementation guidance related to perinatal telebehavioral health care in the U.S. through a comprehensive website and gray literature review, supplemented by semi-structured clinician interviews. This approach was designed to ensure a thorough understanding of existing resources and to highlight areas where guidance may be lacking.

We defined perinatal care as services provided during pregnancy through one year postpartum; telehealth as any remote delivery of healthcare services using telecommunications technology, including video and audio-only visits, electronic consultations, remote patient monitoring, and other digital health tools; and behavioral health as mental health and substance use disorders. These definitions were applied during the screening process to determine resource relevance and inclusion, ensuring that the focus remained on materials pertinent to the needs of perinatal populations.

The scan included resources that referenced telehealth in perinatal populations, regardless of whether the content was behavioral health-specific, to ensure comprehensive identification of available guidance that could inform perinatal telebehavioral health implementation. The sample was assembled iteratively, with some websites identified through targeted Google and gray literature searches combining terms such as “perinatal,” “maternal,” “telehealth,” and “telebehavioral health.” In addition, we consulted with experts in the field to assess the list of organizations and pinpoint any gaps or missing resources.

Two researchers independently reviewed each site between March and June 2025 to identify clinician-facing resources addressing the perinatal period. All identified guidance documents and resources were abstracted into a structured spreadsheet. The research team aggregated and summarized basic document characteristics (e.g., document format, target audience, scope) and categorized the content of each document. Content categorization was focused on perinatal focus, mention of behavioral health, care continuum coverage (i.e., screening and initial assessment, treatment and monitoring, and education), and implementation domains (i.e., payment and coverage, workforce requirements, clinical appropriateness, scheduling, communication, privacy and confidentiality, and technological infrastructure). Patterns and gaps were examined using content analysis. Particular attention was paid to identifying shortcomings in existing guidance that may affect the quality of care provided, including underrepresented populations, care phases, or implementation domains.

To enrich these findings, we conducted 30–45-minute virtual interviews with nine purposively sampled experts representing a range of specialty areas (e.g., psychiatrists, addiction medicine, social workers, obstetrician-gynecologists) and non-clinician roles (e.g., administrators, educator/trainers) involved in perinatal telebehavioral health care. Recruitment continued until no new major themes emerged. Interviews explored experiences implementing perinatal telebehavioral health care and perceptions of available guidance. All interviews were recorded with permission, and detailed notes were taken. A qualitative descriptive approach was applied, with two researchers coding the near-verbatim notes inductively and refining themes through continuous consensus discussion.

## Results

The scan covered 71 organizational websites representing national and federal agencies, regional telehealth resource centers, and relevant state programs (see Table 1). Among the 71 organizations reviewed, we identified 20 (28%) with publicly available perinatal telehealth guidance, and 16 of these addressed perinatal telebehavioral health, yielding 19 unique resources. American College of Obstetricians and Gynecologists (ACOG), the Agency for Healthcare Research and Quality (AHRQ), and the Society for Maternal Fetal Medicine (SMFM) each produced multiple materials. Most resources (14 of 19 resources) offered general telehealth recommendations with only brief references to behavioral health, while five documents (developed by Postpartum Support International, the Maternal Mental Health Alliance, the American Society of Addiction Medicine, and NC MATTERS) were explicitly focused on using telehealth for perinatal behavioral health conditions. Thirteen resources addressed the perinatal period broadly, and six were prenatal-specific; notably, none offered postpartum-specific telebehavioral health guidance. Formats varied, including websites (*n* = 6), reports or issue briefs (*n* = 6), webinars (*n* = 3), tip sheets and infographics (*n* = 2), and clinical consensus statements (*n* = 2). Audiences ranged from clinicians to agencies and policymakers, though intended users were unclear for two resources.

**Table 1:** Organizational websites reviewed (*n* = 71)

Type	List of organizations or associations reviewed
National or Federal Organizations	• Administration for Children and Families (ACF) • Agency for Healthcare Research and Quality (AHRQ) • Alliance for Innovation on Maternal Health (AIM) • American Academy of Family Physicians (AAFP) • American Academy of Pediatrics (AAP) • American Academy of Physician Associates (AAPA) • American Association for Marriage and Family Therapy (AAMFT) • American Association of Nurse Practitioners (AANP) • American College of Nurse Midwives (ACNM) • American College of Obstetricians and Gynecologists (ACOG) • American College of Physicians (ACP) • American Counseling Association (ACA) • American Medical Association (AMA) • American Psychiatric Association (APA) • American Psychological Association (APA) • American Public Health Association (APHA) • American Society of Addiction Medicine (ASAM) • Association for Behavioral and Cognitive Therapies (ABCT) • Association of Maternal and Child Health Programs (AMCHP) • Association of Women’s Health, Obstetrics, and Neonatal Nurses (AWHONN) • Center for Connected Health Policy (CCHP) • Center for Medicare & Medicaid Services (CMS) • Center for Telehealth and e-Health Law (CTeL) • Centers for Disease Control and Prevention (CDC) • Centers for Medicare & Medicaid Services (CMS) • Council on Patient Safety in Women’s Health Care - DISSOLVED IN 2021 • Health Resources and Services Administration (HRSA) • Kaiser Family Foundation (KFF) • Maternal Mental Health Leadership Alliance (MMHLA) • Maternal Mental Health NOW • The American Journal of Maternal/Child Nursing (AJMCN) • National Alliance on Mental Illness (NAMI) • National Association of Certified Professional Midwives (NACPM) • National Association of Social Workers (NASW) • National Birth Equity Collaborative (NBEC) • National Council for Mental Wellbeing • National Council on Alcoholism and Drug Dependence (NCADD) • National Health Law Program (NHeLP) • National Perinatal Association (NPA) • National Telehealth Technology Assessment Resource Center • North American Society for Psychosocial Obstetrics and Gynecology (NASPOG) • Patient-Centered Outcomes Research Institute (PCORI) • Postpartum Support International (PSI) • Rural Health Information (RHI) Hub • Society for Behavioral Medicine (SBM) • Society for Maternal Fetal Medicine (SMFM) • Substance Abuse and Mental Health Services Administration (SAMHSA) • Telehealth Centers of Excellence (COEs) • The Center of Excellence for Protected Health Information (COE-PHI) • The Marcé Society of North America (MONA) • The National Academy for State Health Policy (NASHP) • National Consortium of Telehealth Resource Centers (NCTRC) • Urban Institute (UI)
Regional organizations	• Southwest Telehealth Resource Center (SWTRC) • Great Plains Telehealth Resource and Assistance Center (gpTRAC) • Heartland Telehealth Resource Center (HTRC) • Mid-Atlantic Telehealth Resource Center (MATRC) • Northeast Telehealth Resource Center (NETRC) • Northwest Regional Telehealth Resource Center (NRTRC) • Pacific Basin Telehealth Resource Center (PBTRC) • South Central Telehealth Resource Center (SCTRC) • Southeastern Telehealth Resource Center (SETRC) • TexLa Telehealth Resource Center (TexLa TRC) • Upper Midwest Telehealth Resource Center (UMTRC)
Selected state organizations	• California Maternal Quality Care Collaborative (CMQCC) • California Telehealth Resource Center (CTRC) • Massachusetts Child Psychiatry Access Program for Moms (MCPAP for Moms) • NC MATTERS, UNC Chapel Hill • New York State Department of Health (NY DOH) • Pennsylvania Perinatal Quality Collaborative (PA PQC) • Washington State Perinatal Support Network (WSPC)

The five behavioral health-focused resources covered core implementation domains such as patient screening, treatment initiation and monitoring, telehealth appropriateness, communication strategies, privacy considerations, technological requirements, and evidence-based adaptations. Examples included a billing code infographic, a perinatal psychiatry access website, and webinars referencing privacy and confidentiality, technological requirements, and adapting interventions such as eye movement desensitization and reprocessing (EMDR) to a telehealth context.

Clinician interviews, however, revealed that existing guidance does not address several concrete clinical realities of perinatal telebehavioral health care. Clinicians described uncertainty about how to administer validated screening tools such as the Edinburgh Postnatal Depression Scale during virtual visits, with one asking, “What’s the best way of filling out the Edinburgh scale…do you hold it up? Do you give it to them ahead of time?” Several pointed to persistent misinformation about pharmacologic management during pregnancy and lactation, particularly for opioid use disorder (OUD): “When it comes to OUD treatment, there’s still a lot of misinformation, even among practicing clinicians, about…what can be safely done in a lot of cases via telehealth compared to in-person.” Clinicians also described the unique challenge of managing the dyadic nature of perinatal care during virtual sessions, both the clinical opportunity to observe parent-infant interaction and feeding via video and the difficulty of conducting sensitive conversations when older children are present. As one clinician observed, “if mom has a newborn and they’re breastfeeding, that’s fine to be in that [telehealth] session, but a one- or two-year-old is not appropriate when we’re talking about trauma.” Substance use raised additional complexity, as state-mandated reporting requirements vary widely and are often poorly understood by both clinicians and patients, with potential consequences including referrals to Child Protective Services. Across these areas, clinicians reported relying on experiential learning or institutional policies developed ad hoc, emphasizing the absence of standardized, perinatal‑specific telebehavioral health guidance.

## Discussion

This environmental scan highlights major gaps in implementation guidance for telebehavioral health care during the perinatal period. While general telehealth resources are plentiful, only about one in four relevant U.S.-based organizations provided any perinatal‑specific telehealth materials, and even fewer specifically addressed behavioral health. Much of what exists emerged rapidly during the COVID‑19 public health emergency to help clinicians navigate new reimbursement structures and virtual‑care workflows. Although these resources were valuable in facilitating rapid adoption, few have been updated to reflect the post‑pandemic policy environment or the unique clinical and social considerations of perinatal patients.

The gaps identified reflect deeper structural issues. Perinatal behavioral health care sits at the intersection of obstetrics, psychiatry, addiction medicine, pediatrics, primary care and social work, yet existing guidance rarely bridges these silos. Few resources reflect input from perinatal psychiatry specialists, and policy attention to perinatal behavioral health has lagged behind investment in either telehealth or maternal health more broadly. As a result, clinicians are left to translate general telehealth principles into a clinical context that involves rapid physiologic change, evolving medication considerations across pregnancy and lactation, mandated reporting obligations that vary by state, and the simultaneous care of a parent and infant.

The absence of postpartum‑specific telebehavioral health guidance is particularly striking. The postpartum year is a period of heightened psychiatric vulnerability, with opioid overdose and suicide risks peaking 7–12 months after delivery (Schiff et al. 2018)—precisely the window during which many patients lose continuous insurance and routine perinatal clinician contact. Telehealth is well positioned to bridge this gap by lowering barriers to engagement, yet existing resources offer little practical direction on virtual postpartum screening, dyadic assessment, medication management during lactation, or coordinated handoffs between obstetric and behavioral health clinicians. Without targeted guidance, postpartum patients risk “falling through the cracks” that telehealth can help close.

While telehealth has the potential to reduce access barriers and promote equity, the lack of clear implementation guidance limits its impact and may reinforce disparities across systems and populations. Rural patients face persistent broadband limitations; lower-income patients may lack private space, devices, or unlimited data; and patients with limited digital literacy—including some adolescent and immigrant populations—may struggle to navigate telehealth platforms even when access is technically available. Reimbursement asymmetries between video and audio-only visits further disadvantage the patients most likely to rely on the latter. These structural realities mean that, in the absence of equity-focused implementation guidance, telehealth expansion may widen rather than narrow gaps in perinatal behavioral health care.

Drawing on our scan and interview findings, we offer several recommendations for the development of future perinatal telebehavioral health guidance. First, guidance should provide concrete protocols for remote administration of validated screening tools such as the EPDS, including whether to send instruments in advance, how to score in real time, and how to manage disclosures during virtual visits. Second, guidance should address privacy considerations specific to the perinatal context, where private space is often limited by the presence of a newborn, older children, or other household members. Third, guidance should offer practical strategies for positioning and engaging the infant on camera to enable dyadic assessment, feeding observation, and parent–infant interaction. Fourth, guidance should support coordination and warm handoffs with primary care providers, obstetricians, and pediatricians, who are often the first points of contact for perinatal patients. Fifth, guidance should clarify evidence-based approaches to pharmacologic management during pregnancy and lactation, including buprenorphine and methadone for opioid use disorder, to counter persistent misinformation about what can safely be delivered via telehealth versus in-person care. Together, these elements would help clinicians realize the full potential of perinatal telebehavioral health care.

## Limitations

Several limitations should be considered when interpreting these findings. First, this scan was limited to U.S.-based organizations and publicly available, English-language guidance, reflecting the country-specific nature of telehealth regulation, reimbursement, licensure, and mandated reporting requirements. Second, despite iterative searching and expert consultation, relevant guidance hosted in subscription-restricted, non-indexed, or non-English venues may have been missed; podcasts and other formats were also outside our review scope. Third, our interview sample, while purposively constructed for diverse clinical and non-clinical expertise, was modest in size and drew from a limited number of states. Perspectives from clinicians in additional regions (e.g., highly rural areas and clinicians with less advanced telehealth infrastructure) may provide additional context to the gaps identified here.

## Future directions

Future efforts should prioritize the development of concise, evidence-based perinatal telebehavioral health guidance that addresses the clinical and structural gaps identified above. Achieving this will require collaboration among professional societies, telehealth resource centers, perinatal psychiatry access programs, and state Medicaid agencies, with explicit attention to the postpartum year and to populations at greatest risk of being left behind. Subsequent work should also draw on international models, including those from settings with advanced rural and remote tele-mental health infrastructure, to inform U.S. guidance development. Lessons from telehealth deployment in disaster- and conflict-affected settings, where in-person perinatal behavioral health services are frequently disrupted, may further inform the development of resilient and adaptable implementation guidance. Developing tailored, accessible resources is an essential next step toward fully realizing telehealth’s potential to improve maternal mental health and reduce maternal morbidity and mortality.

## Data Availability

All data generated or analyzed during this study are included in this published article and its supplementary information files.

## References

[CR1] Aboujaoude E, Salame W, Naim L (2015) Telemental health: A status update. World Psychiatry 14:223–230. 10.1002/wps.20218 PMC4471979 26043340

[CR2] American College of Obstetricians and Gynecologists (2020) Implementing telehealth in practice. Obstet Gynecol 135:e73–79. 10.1097/AOG.0000000000003672

[CR3] American College of Obstetricians and Gynecologists (n.d.-a) Perinatal mental health. Accessed 10 Sep 2025. https://www.acog.org/programs/perinatal-mental-health

[CR4] American Psychiatric Association (2023) What is perinatal depression?. Accessed 23 Jul 2025. https://www.psychiatry.org/patients-families/Peripartum-Depression/What-is-Peripartum-Depression

[CR5] Batastini AB, Paprzycki P, Jones ACT, MacLean N (2021) Are videoconferenced mental and behavioral health services just as good as in-person? A meta-analysis of a fast-growing practice. Clin Psychol Rev 83:101944. 10.1016/j.cpr.2020.101944 33227560

[CR6] Brown CC, Adams CE, George KE et al (2021) Mental health conditions increase severe maternal morbidity by 50% and cost $102 million yearly in the United States. Health Aff (Millwood) 40:1575–1584. 10.1377/hlthaff.2021.00759 PMC8759410 34606352

[CR24] Carlson K, Mughal S, Azhar Y, Siddiqui W (2025) "Perinatal Depression." StatPearls, StatPearls Publishing, 22 Jan. https://www.ncbi.nlm.nih.gov/books/NBK519070/30085612

[CR7] Center for Connected Health Policy (2024) State telehealth laws and reimbursement policies report, fall 2024. Accessed 9 Sep 2025. https://www.cchpca.org/resources/state-telehealth-laws-and-reimbursement-policies-report-fall-2024/

[CR8] Centers for Disease Control Prevention (2026) Pregnancy-Related Deaths: Data from Maternal Mortality Review Committees. https://www.cdc.gov/maternal-mortality/php/data-research/mmrc/index.html?cove-tab=1

[CR9] Declercq E, Feinberg E, Belanoff C (2022) Racial inequities in the course of treating perinatal mental health challenges: Results from listening to mothers in California. Birth 49:132–140. 10.1111/birt.12584 PMC9292331 34459012

[CR10] Hilty DM, Ferrer DC, Parish MB et al (2013) The effectiveness of telemental health: A 2013 review. Telemed J E Health 19:444–454. 10.1089/tmj.2013.0075 PMC3662387 23697504

[CR11] Huggins B, Jones C, Adeyinka O et al (2020) Racial disparities in perinatal mental health. Psychiatr Ann 50:489–493. 10.3928/00485713-20201007-02

[CR12] Kissler K, Thumm EB, Smith DC, Anderson JL (2024) Perinatal telehealth: Meeting patients where they are. J Midwifery Women’s Health 69:9–16. 10.1111/jmwh.13560 PMC10873126 37641584

[CR13] Koonin LM, Hoots B, Tsang CA et al (2020) Trends in the use of telehealth during the emergence of the COVID-19 pandemic — United States, January–March 2020. MMWR Morb Mortal Wkly Rep 69:1595–1599. 10.15585/mmwr.mm6943a3 PMC7641006 33119561

[CR14] Kozhimannil KB, Trinacty CM, Busch AB et al (2011) Racial and ethnic disparities in postpartum depression care among low-income women. Psychiatr Serv 62:619–625. 10.1176/appi.ps.62.6.619 PMC3733216 21632730

[CR15] Landis RK, Stein BD, Dick AW et al (2023a) Trends and disparities in perinatal opioid use disorder treatment in Medicaid, 2007–2012. Med Care Res Rev. 10.1177/10775587231216515 PMC12931483 38160405

[CR16] Landis RK, Stein BD, Griffin BA et al (2023b) Disparities in perinatal and emergency care receipt among women with perinatal opioid use disorder in Medicaid, 2007 to 2012. J Addict Med 17:654–661. 10.1097/ADM.0000000000001199 PMC10759200 37934525

[CR17] Maternal Mental Health Leadership Alliance (2024) Maternal mental health conditions and statistics: An overview. Accessed 9 Sep 2025. https://www.mmhla.org/articles/maternal-mental-health-conditions-and-statistics

[CR18] Mohr DC, Ho J, Duffecy J (2012) and al e Effect of telephone-administered vs face-to-face cognitive behavioral therapy on adherence to therapy and depression outcomes among primary care patients: A randomized trial. JAMA 307:2278–2285. 10.1001/jama.2012.5588 PMC3697075 22706833

[CR19] Mukherjee S, Trepka MJ, Pierre-Victor D et al (2016) Racial/ethnic disparities in antenatal depression in the United States: A systematic review. Matern Child Health J 20:1780–1797. 10.1007/s10995-016-1989-x 27016352

[CR20] Schiff D, Neilson T, Terplan M, Hood M (2018) Fatal and nonfatal overdose among pregnant and postpartum women in Massachusetts. Obstet Gynecol 132:466–474. 10.1097/AOG.0000000000002734 PMC6060005 29995730

[CR21] Sidebottom A, Vacquier M, LaRusso E et al (2021) Perinatal depression screening practices in a large health system: identifying current state and assessing opportunities to provide more equitable care. Arch Womens Ment Health 24:133–144. 10.1007/s00737-020-01035-x PMC7929950 32372299

[CR22] Sujan AC, Nance N, Quesenberry C et al (2023) Racial and ethnic differences in perinatal depression and anxiety. J Affect Disord 334:297–301. 10.1016/j.jad.2023.04.123 PMC10234114 37156281

[CR23] Vesga-López O, Blanco C, Keyes K et al (2008) Psychiatric disorders in pregnant and postpartum women in the United States. Arch Gen Psychiatry 65:805–815 10.1001/archpsyc.65.7.805 PMC2669282 18606953

